# Comprehensive Analysis of Prognostic Microenvironment-Related Genes in Invasive Breast Cancer

**DOI:** 10.3389/fonc.2021.576911

**Published:** 2022-01-03

**Authors:** Yingrong Shi, Si Chen, Huijuan Xing, Guanglie Jiang, Nan Wu, Qiannan Liu, Norihiro Sakamoto, Takayoshi Kuno, Reiko Sugiura, Qinghuan Xiao, Feng Jin, Yue Fang, Fan Yao

**Affiliations:** ^1^ Department of Microbial and Biochemical Pharmacy, School of Pharmacy, China Medical University, Shenyang, China; ^2^ Division of Food and Drug Evaluation Science, Kobe University Graduate School of Medicine, Kobe, Japan; ^3^ Laboratory of Molecular Pharmacogenomics, School of Pharmaceutical Sciences, Kinki University, Higashi-Osaka, Japan; ^4^ Department of Ion Channel Pharmacology, School of Pharmacy, China Medical University, Shenyang, China; ^5^ Department of Breast Surgery and Surgical Oncology, Research Unit of General Surgery, The First Affiliated Hospital of China Medical University, Shenyang, China

**Keywords:** invasive breast cancer, TCGA database, prognosis, SASH3, immune/stromal/ESTIMATE score, tumor microenvironment

## Abstract

Recent studies reveal that tumor microenvironment contributes to breast cancer (BRCA) development, progression, and therapeutic response. However, the contribution of the tumor microenvironment-related genes in routine diagnostic testing or therapeutic decision making for BRCA remains elusive. Immune/stromal/ESTIMATE scores calculated by the ESTIMATE algorithm quantify immune and stromal components in a tumor, and thus can reflect tumor microenvironment. To investigate the association of the tumor microenvironment-related genes with invasive BRCA prognosis, here we analyzed the immune/stromal/ESTIMATE scores in combination with The Cancer Genome Atlas (TCGA) database in invasive BRCA. We found that immune/stromal/ESTIMATE scores were significantly correlated with the invasive BRCA clinicopathological factors. Based on the immune/stromal/ESTIMATE scores, we extracted a series of differential expression genes (DEGs) related to the tumor microenvironment. Survival analysis was further performed to identify a list of high-frequency DEGs (HF-DEGs), which exhibited prognostic value in invasive BRCA. Importantly, consistent with the results of bioinformatics analysis, immunohistochemistry results showed that high SASH3 expression was associated with a good prognosis in invasive BRCA patients. Our findings suggest that the tumor microenvironment-related HF-DEGs identified in this study have prognostic values and may serve as potential biomarkers and therapeutic targets for invasive BRCA.

## Introduction

Breast cancer (BRCA) is one of the most common cancers worldwide and is the second leading cause of cancer-related mortality in women. The mortality rate of BRCA is expected to increase in the next few years, especially in the developing countries (1). Although considerable progress has been made in the clinical treatment of BRCA, there are still many patients with poor prognosis. Therefore, identification of more accurate and reliable biomarkers is important for early diagnosis and individualized treatment of BRCA.

In recent years, the clinical diagnosis for cancer, especially the accurate molecular diagnosis, has been more and more based on its gene expression. The Cancer Genome Atlas (TCGA) database offers a comprehensive, multidimensional map of genomic data with 33 cancer types including BRCA. It has been widely applied for identifying tumor biomarkers and cancer-specific signatures (2).

The tumor microenvironment plays an important role in the occurrence and development of cancer. It has been reported that the tumor microenvironment affects BRCA development, growth, migration, metastasis, and treatment resistance (3). The tumor microenvironment consists of not only proliferating tumor cells but also surrounding stromal cells and infiltrating immune cells as well as many secreted factors (4). Immune cells and stromal cells, the two main types of nontumor components in the tumor microenvironment, are valuable in the diagnosis and prognosis of tumors (4). Yoshihara et al. developed the Estimation of Stromal and Immune cells in Malignant Tumor tissues using Expression data (ESTIMATE) algorithm, which calculated immune/stromal/ESTIMATE scores using gene expression data from the TCGA database (5). The ESTIMATE algorithm can predict the infiltration of nontumor cells in malignant tumor tissues and assess the tumor purity. This algorithm has been initially applied to many cancers, such as prostate cancer, BRCA, colon cancer, glioblastoma, and so on (6). However, the effectiveness and applicability of immune/stromal/ESTIMATE scores in BRCA, especially identification of invasive BRCA prognostic genes using stromal/immune/ESTIMATE scores, has not been investigated in detail.

In this study, we extracted a list of tumor microenvironment-related high-frequency differential expression genes (HF-DEGs) in the genome-wide level by comprehensively analyzing the invasive BRCA gene-expression profile and clinical information in TCGA database as well as invasive BRCA immune/stromal/ESTIMATE scores calculated by ESTIMATE algorithm. Notably, the HF-DEGs identified here exhibited important prognostic values and may be promising biomarkers for invasive BRCA.

## Materials and Methods

### Data Sources

The gene expression profile (AgilentG4502A expression data) for invasive BRCA patients was obtained from the TCGA data portal (https://tcga-data.nci.nih.gov/tcga/). The relevant clinical information was downloaded and analyzed, including age, gender, hormone receptor expression status, pathological stage, histological grade, lymph node status, molecular subtypes, treatment method, relapse, and survival. The data collection and application were performed in accordance with the TCGA publication guidelines and data access policies.

The ESTIMATE algorithm developed by Yoshihara et al. can calculate immune/stromal/ESTIMATE scores to output the estimated levels of infiltrating immune and stromal cells as well as estimated tumor purity by using gene expression data from the TCGA database (5). Briefly, the immune/stromal/ESTIMATE score analysis is mainly based on two gene signatures: a “stromal signature” that was designed to capture the presence of stromal in tumor tissue, and an “immune signature” that aimed to represent the infiltration of immune cells in tumor tissue (5). By calculating the expression levels of stromal signature and immune signature in breast cancer patients, the stromal score and immune score of the corresponding patient can be obtained. The ESTIMATE score can be obtained by the combination of stromal score and immune score (5). The ESTIMATE algorithm is publicly available through the SourceForge software repository (https://sourceforge.net/projects/estimateproject), and all the immune/stromal/ESTIMATE scores of invasive breast cancer patients calculated by the ESTIMATE algorithm (based on AgilentG4502A expression data) were downloaded from https://bioinformatics.mdanderson.org. Invasive BRCA cases were classified into the high- and low-score groups based on the median value of the immune/stromal/ESTIMATE scores.

A total of 481 patients with immune/stromal/ESTIMATE scores and clear clinicopathological factors were used to analyze whether the invasive BRCA clinicopathological factors were related to immune/stromal/ESTIMATE scores. Unpaired *t*-test was used to compare immune/stromal/ESTIMATE scores for different kinds of clinicopathological factors, while ordinary one-way ANOVA test were used to carry out the relationship between molecular subtypes and immune/stromal/ESTIMATE scores. In total, 488 patients containing immune/stromal/ESTIMATE scores as well as gene expression profile were used to identify the differentially expressed genes which related to the tumor microenvironment. Furthermore, 334 patients with clear prognosis and invasive BRCA gene expression profile were used for survival analysis. Lastly, 467 patients with invasive BRCA gene expression profile as well as detailed clinical information were used for COX analysis.

### Data Processing

Package limma was used to screen DEGs with fold change >1.5 and adjusted. *p*-values <0.05. The obtained DEGs were used to draw the volcano graph with log2 (fold change) as the abscissa and the negative logarithm of the *p*-value of the *t*-test [−log10 (*p*-value)] as the ordinate, by using the ggplot2 package in R. The Venn diagrams of DEGs were plotted online (https://bioinfogp.cnb.csic.es). Functional enrichment analysis of DEGs was performed by The Database for Annotation, Visualization and Integrated Discovery (DAVID) to identify GO categories by their biological processes (BP), molecular functions (MF), or cellular components (CC). False discovery rate (FDR) <0.05 was used as the cutoff value.

A protein-protein interaction (PPI) network was constructed using the STRING and reconstructed by Cytoscape software. In addition, the PPI network of overlapping DEGs was obtained from STRING with medium confidence >0.4 as a cutoff criterion. The Molecular Complex Detection (MCODE) plugin in Cytoscape was then used to find the significant modules based on the topology to locate densely connected regions. The settings of selection were as follows: degree cutoff = 2, node score cutoff = 0.2, k-core = 2, and maximum depth = 100. The significant modules with 20 or more nodes were selected for further analysis.

The GlueGo (V2.5.4) plug-in in Cytoscope was used to perform GO biological process analysis and Kyoto Encyclopedia of Genes and Genomes (KEGG) pathway enrichment on the DEGs contained in each module and the HF-DEGs. The ClueGo settings of module 1 to module 4 selections were as follows: Ontologies/Pathways = GO-Biological Process/KEGG, Evidence = All, Advanced Term/Pathway selection options: GO Tree Interval: Min = 1, Max = 3, GO Tree/Pathway Network Connectivity = 0.5, others are the default values. The ClueGo settings of the HF-DEGs selections were as follows: Ontologies/Pathways = GO-Biological Process/KEGG, Evidence = All, Advanced Term/Pathway selection options: GO Tree Interval: Min = 3 Max = 8, GO Tree/Pathway Network Connectivity = 0.4, others are the default values.

Kaplan-Meier survival analysis was performed to identify DEGs associated with BRCA prognosis using log-rank tests in R. *p*-values of <0.05 were considered statistically significant. The ggsurvplot function in the R was used to draw the RFS curve of patients in the TCGA database. The mapping function was used to draw the RFS curve of BRCA patients on the Kaplan-Meier Plotter (kmplot.com).

### Patient Tissue Samples

Formalin-fixed, paraffin-embedded invasive breast tumors (*n* = 172), and the corresponding clinicopathological data were obtained from the Department of Breast Surgery at the First Affiliated Hospital of China Medical University. Patient’s data included lymph node status, age, tumor size, hormone receptor expression status, and time to relapse. This study was approved by the Medical Ethics Committee of China Medical University, and the need of written informed consent by the patients was waived due to the retrospective nature of the study. The study was carried out according to the Declaration of Helsinki, which was approved by the Scientific Ethical Committee of China Medical University (No. 2017066).

### Immunohistochemistry

Human invasive BRCA tissue sections (4 μm thick) with human invasive BRCA tissue were used for immunohistochemistry. Briefly, sections were cut, paraffin removed, rehydrated, and washed in phosphate-buffered saline (PBS). After antigen recovery, the sections were incubated with primary antibodies against SASH3 (1:100 dilution; Abcam Biotechnology, Cambridge, UK) overnight at 4°C. The sections were then incubated with biotinylated secondary antibodies (1:3,000 dilution; UltraSensitiveTM-SP kit, Fuzhou Maixin, Fuzhou, China) and horseradish peroxidase-coupled streptavidin for 20 min. The sections were then incubated with 3,3-diaminobenzidine for 2 min.

Two researchers evaluated the results of immunohistochemical staining under light microscope. Scoring of the expression was performed semiquantitatively as consensus report of the task force for basic research of the EORTC-GCCG (European Organization for Research and Treatment of Cancer-Gynaecological Cancer Cooperative Group) (7). Weak/moderate staining with positive cell percentage <1% or no staining were defined as 0, weak/moderate staining with positive cell percentage ≤5% were defined as 1, weak/moderate staining with positive cell percentage ≤30% were defined as 2, and weak/moderate staining with a positive cell percentage >30% or strong staining with any positive cell percentage were defined as 3. The results were subsequently dichotomized for statistical analysis, and the defined cutoff point of high expression for the statistical analysis was set to 2.

### Statistical Analyses

Statistical analyses were performed by using SPSS (IBM SPSS Statistics 22.0, Inc., Chicago, IL, USA). The log-rank test was used to identify the association between SASH3 expression and BRCA prognosis. *p*-values <0.05 were considered statistically significant.

## Results

### The Correlation Between Immune/Stromal/ESTIMATE Scores and Clinicopathological Factors in Invasive Breast Cancer

Liu et al. have reported that the BRCA patients with higher immune/stromal scores were associated with longer overall survival time (OS) (6), suggesting that immune/stromal scores may be used as a prognostic biomarker for BRCA. We then investigated whether not only the immune/stromal scores but also the ESTIMATE scores were correlated with the clinicopathological factors in invasive BRCA. The results showed that the clinicopathological factors of molecular subtypes, The American Joint Committee on Cancer (AJCC) stage, tumor size, ER status, PR status, and HER2 status were significantly correlated with all the immune/stromal/ESTIMATE scores in invasive BRCA (*p* < 0.05, **Table 1**). In addition, the clinicopathological factors of age, metastasis, and number of lymph nodes were significantly correlated with ESTIMATE scores in invasive BRCA (age: *p* < 0.0001, metastasis: *p* = 1.26E−07, lymph node: *p* < 0.0001, **Table 1**).

**Table 1:** Distribution of invasive breast cancer patients’ characteristics and their correlation with stromal/immune/ESTIMATE scores.

Variables	Count (total *n* = 481)	Stromal score	Immune score	ESTIMATE score
		*p*-values	*p*-values	*p*-values
**Molecular subtypes**				
** Normal-like**	7	0.003^**^	<0.0001^***^	0.0072^**^
** Luminal B**	119			
** Luminal A**	209			
** HER2-enriched**	56			
** Basal-like**	90			
**Age**				
** <60**	247	0.0994	0.1220	<0.0001^***^
** ≥60**	234			
**Metastasis**				
** Negative**	468	0.9672	0.1346	1.26E−07^***^
** Positive**	13			
**Lymph nodes**				
** Negative**	238	0.5445	0.1591	<0.0001^***^
** Positive**	243			
**AJCC stage**				
** I–II**	356	1.02E−14^***^	<0.0001^***^	1.13E−07^***^
** III–V**	125			
**Tumor size**				
** T1–T2**	405	1.50E−11^***^	3.29E−09^***^	<0.0001^***^
** T3–T5**	76			
**ER**				
** Negative**	365	2.96E−12^***^	<0.0001^***^	<0.0001^***^
** Positive**	116			
**PR**				
** Negative**	315	<0.0001^***^	<0.0001^***^	<0.0001^***^
** Positive**	173			
**HER2**				
** Negative**	418	2.06E−09^***^	5.91E−11^***^	<0.0001^***^
** Positive**	70			

The bold terms are significant variables. ^**^p < 0.01, ^***^p < 0.001.

### The Correlation Between Invasive Breast Cancer Gene Expression and Immune/Stromal/ESTIMATE Scores

**Figure 1:**
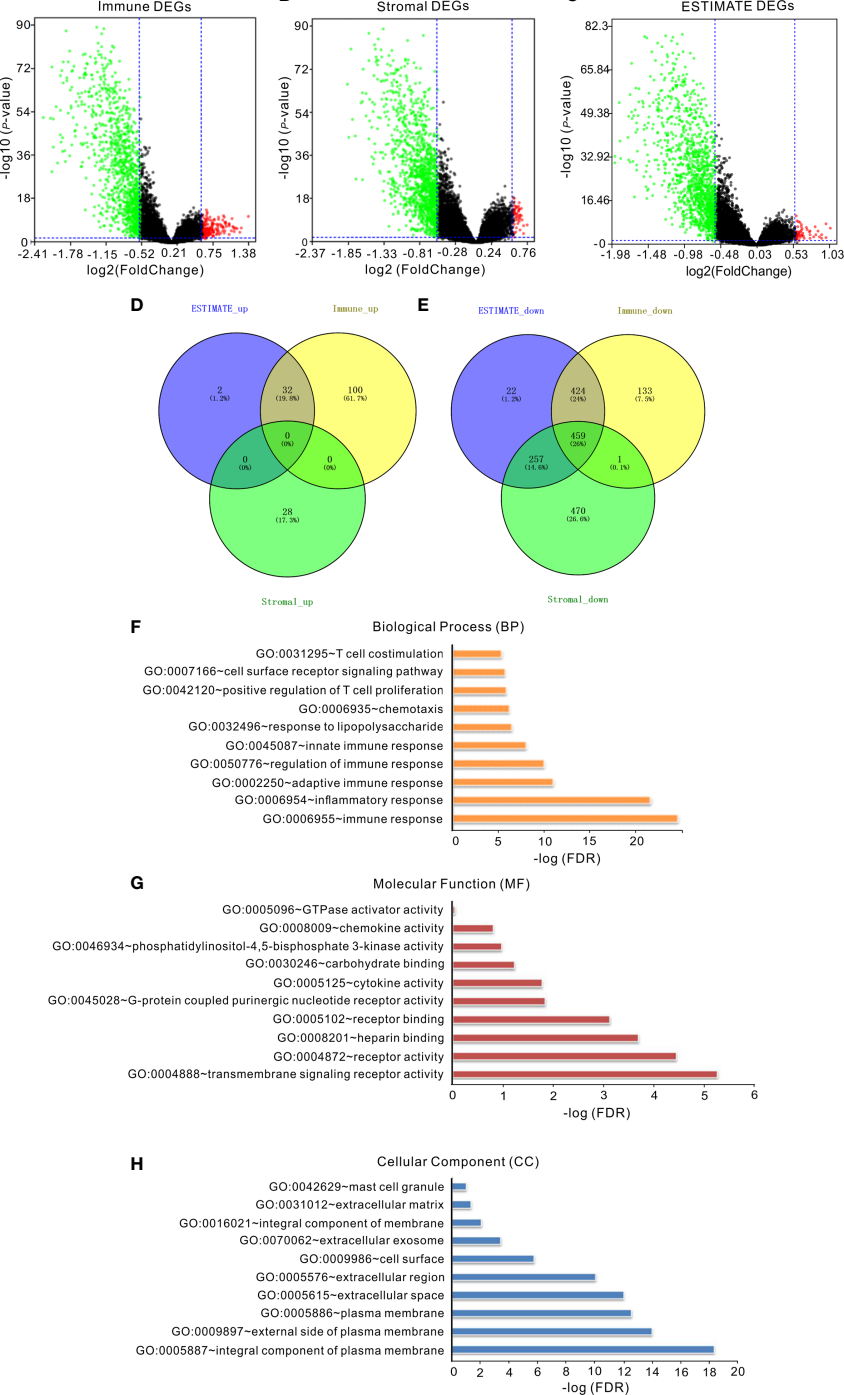
The correlation of gene expression with immune/stromal/ESTIMATE scores. **(A)** Volcano map of DEGs of immune scores. **(B)** Volcano map of DEGs of stromal scores. **(C)** Volcano map of DEGs of ESTIMATE scores. Genes with higher expression are shown in red; lower expression are shown in green; genes with same expression level are shown in black (*p* < 0.05, log2 (FC) >2). **(D, E)** Venn diagrams showed the number of upregulated **(D)** or downregulated **(E)** DEGs in stromal/immune/ESTIMATE scores groups. **(F–H)** The top 10 GO terms of overlapping DEGs. False discovery rate (FDR) of GO analysis was acquired from DAVID functional annotation tool (*p* < 0.05).

To reveal the correlation between gene expression and immune/stromal/ESTIMATE scores, we analyzed the gene expression profile of 488 invasive BRCA patients in the TCGA database. Volcano map showed that a total of 1,149 differential expression genes (DEGs) were identified, including 132 upregulated genes and 1,017 downregulated genes in the high immune-score group when compared with the low immune-score group (fold change >1.5, *p* < 0.05, **Figure 1A**). Likewise, a total of 1,215 DEGs were identified with 28 upregulated genes and 1,187 downregulated genes in the high stromal score group when compared with the low stromal score group (fold change >1.5, *p* < 0.05, **Figure 1B**). In addition, a total of 1,196 DEGs were identified with 34 upregulated genes and 1,162 downregulated genes in the high ESTIMATE score group when compared with the low ESTIMATE score group (fold change >1.5, *p* < 0.05, **Figure 1C**). Moreover, as shown in the Venn diagrams (**Figures 1D, E** **)**, 459 genes were downregulated in all the groups of the immune score group, the stromal score group, and the ESTIMATE score group (**Figure 1E**), but no gene was upregulated in all of the three groups (**Figure 1D**). We further performed gene ontology (GO) analysis of the 459 overlapping DEGs, and the top 10 GO terms in biological process, molecular function, and cellular component categories were shown in **Figures 1F–H**. The results showed that the biological processes of these genes were mainly involved in immunity, such as immune response (**Figure 1F**), suggesting that the 459 overlapping DEGs may play an important role in the tumor microenvironment of invasive BRCA. Thus, these 459 overlapping genes were further investigated as follows.

### Protein-Protein Interactions Among DEGs

**Figure 2:**
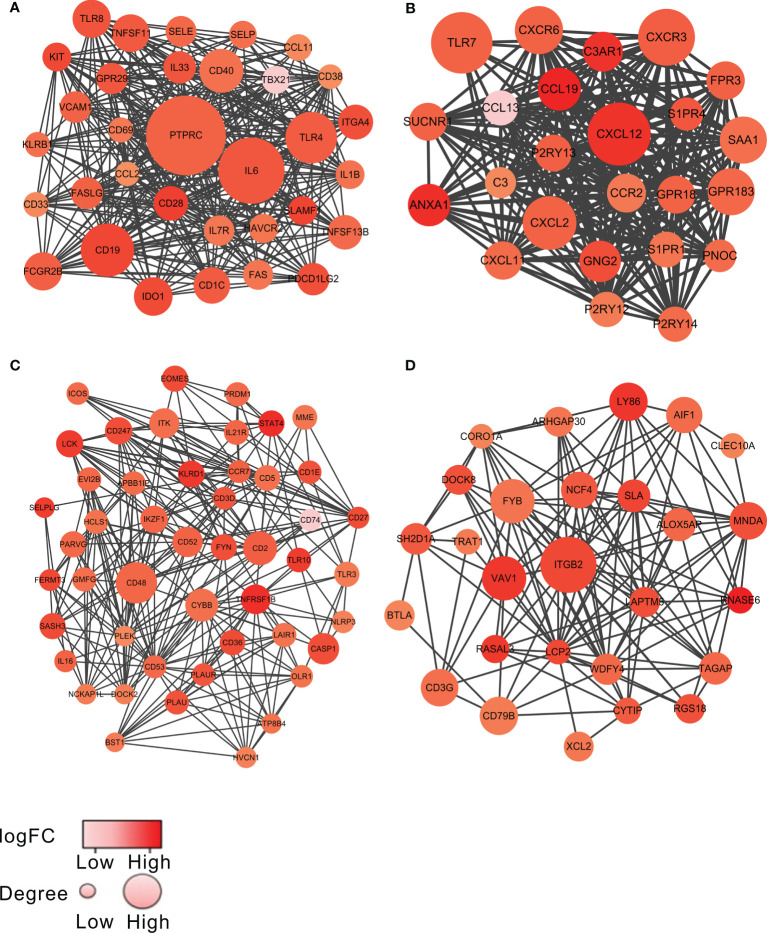
The top 4 significant modules from PPI network. The top 4 modules, named as module 1 to module 4, are shown in **(A–D)**, respectively. The color of a node in the PPI network reflected the log (FC) value of the Z-score of gene expression, and the size of node indicated the number of interacting proteins with the designated protein. The darker color or the larger volume of a node, the more important the node was in each model.

To investigate the potential links between the DEGs, we constructed the PPI network of the 459 overlapping genes by the STRING tool. We then used the MCODE plugin in Cytoscape to identify the significant modules. The network consisted of 8 modules, including 173 nodes and 1,107 edges. The top 4 modules with node number >20 were selected for further analysis (**Figure 2** and **Supplementary Table S1**). For convenience, we named these modules as module 1 to module 4, respectively. As shown in **Figure 2** and **Supplementary Table S1**, the remarkable nodes of each model were obtained. In module 1 (**Figure 2A**), PTPRC had the most connections with other members of the module. Similarly, the remarkable nodes of the other three modules were CXCL12, CD48, and ITGB2, respectively (**Figures 2B–D**).

To explore the biological functions of the genes in each module, we further used the Glue Go plug-in in Cytoscope to perform GO biological process analysis and KEGG pathway enrichment analysis on the DEGs in each module. The GO biological process analysis showed that the DEGs in module 1, module 3, and module 4 were involved in various biological processes, where they were significantly correlated with immune responses, such as immunoglobulin production in module 1 (**Supplementary Figures S1A, B** **)**, thymic T-cell selection in module 3 (**Supplementary Figures S1E, F** **)**, and positive regulation of T-cell migration in module 4 (**Supplementary Figures S1G, H** **)**. On the other hand, the DEGs in module 2 were also enriched in several biological processes including immune responses, such as lymphocyte migration (**Supplementary Figures S1C, D** **)**. Consistent with the results of GO analysis, many pathways yielded from the KEGG analysis on the DEGs of each module showed a close correlation with immune response. The genes in module 1, for example, were associated with intestinal immune network for IgA production (**Supplementary Figures S2A, B** **)**, the genes in module 2 were associated with NF-kappa B signaling pathway (**Supplementary Figures S2C, D** **)**, and the genes in module 3 were associated with T-cell receptor signaling pathway and Th1 and Th2 cell differentiation (**Supplementary Figures S2E, F** **)**.

### Relationship Between DEGs and Prognosis of Invasive Breast Cancer

To determine whether the 459 overlapping DEGs were correlated with the prognosis of invasive BRCA, we analyzed the relapse-free survival time (RFS) of each DEG. Among the 459 overlapping DEGs, a total of 61 DEGs were significantly correlated with the prognosis of invasive BRCA (*p* < 0.05). The RFS results of all DEGs are shown in the **Supplementary Material** (**Supplementary Figure S3**).

To investigate whether the DEGs identified here from the TCGA database have potential prognostic values in other BRCA cases, we further analyzed the RFS of the 459 overlapping genes using another online survival analysis tool, Kaplan-Meier Plotter kmplot.com (8). Among these genes, 392 genes were shown to significantly associate with prognosis prediction (**Supplementary Table S2**, *p* < 0.05). Importantly, 55 DEGs were identified from both the TCGA database and Kaplan-Meier Plotter kmplot.com website (**Table 2**; **Supplementary Table S3**). Thus, these 55 DEGs were named as HF-DEGs, and their biological functions are summarized in **Table 2**.

**Table 2:** The biological functions of HF-DEGs.

Categories	Gene symbols
Receptor	**IL18RAP**, PLAUR, CD40, TNFRSF17, FASLG, CD69, TLR10, **GPR171**, C3AR1, **P2RY12**, DARC, **SUCNR1**, **FPRL2**
Cytokine	IL1B, FIGF, GBP1
Chemokine	CXCL2, CCL11
Nucleotide exchange factor	**RASGRP2**, VAV1, **DOCK2**
Adaptor protein	DOK2, ACSL5
Transcription factor	STAT4, **MDFIC**, TRIM22
Activator or inhibitor activity	**RGS18**, RARRES1, **RUBCNL, CST7**
Signal transmission/transmembrane/skeletal/extracellular matrix/transport protein	**HAVCR2**, TSPAN2, **ADD3**, **WDFY4, DPT**, EMILIN2, **ATP8B4**, **SLC7A7**
Blood coagulation factor	**FGL2**
Complement	C3
GTP- and nucleotide-binding proteins	**GIMAP8**, **GIMAP2**
Enzyme	HSD11B1, NCF4, GZMH, HTRA4, ENPP2, BTK IRAK3, INDO
Unknown function	**CXorf21**, **SAMD3**, **FAM30A**, SASH3, **TMEM100**

Genes in bold have not been previously reported for their prognostic value in invasive breast cancer patients.

**Figure 3:**
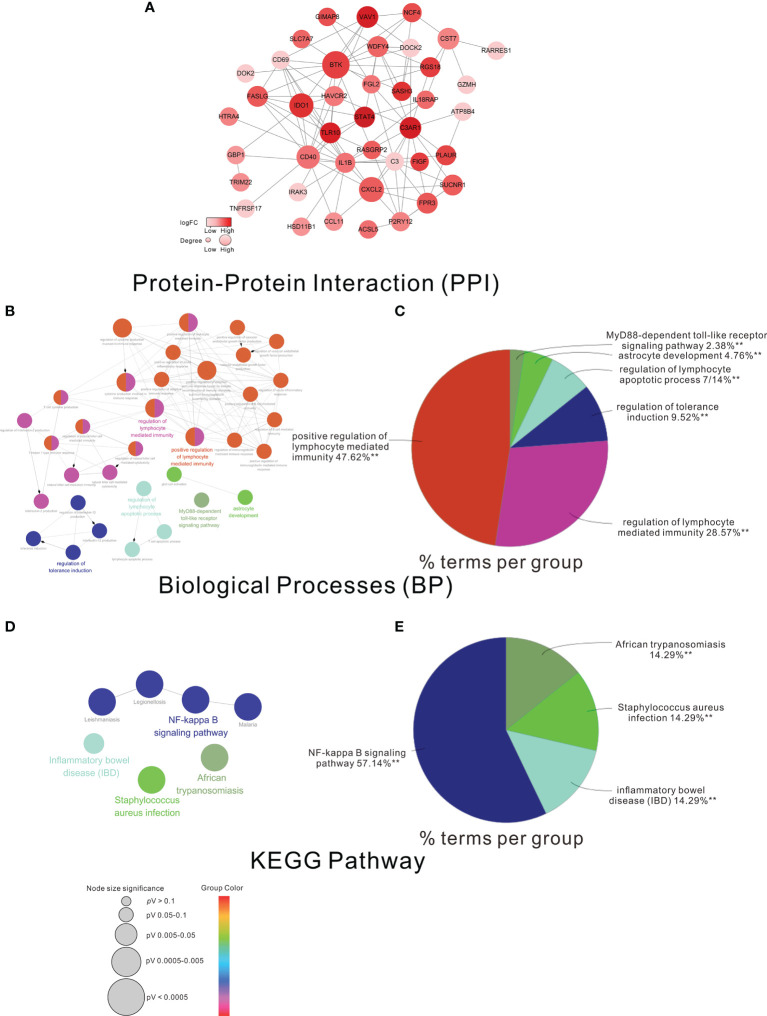
The PPI network GO analysis and KEGG analysis of the HF-DEGs. **(A)** The PPI network of the HF-DEGs. The color of a node in the PPI network reflected the log (FC) value of the Z-score of gene expression, and the size of node indicated the number of interacting proteins with the designated protein. In this PPI network, the log (FC) of the nodes >1.5 was considered the more important nodes. Functional grouped network diagram with GO terms and the KEGG pathways as nodes linked based on the HF-DEGs is shown in **(B, D)**, respectively. The pie charts of **(C, E)** summarized the GO terms and KEGG terms corresponding to the network diagram in **(B, D)**, respectively. The size of node indicated the *p*-value of the biological processes or the KEGG pathway. The color of node indicated the term of the biological processes or the KEGG pathway.

To further understand the functions of the HF-DEGs, we constructed the PPI network of these 55 HF-DEGs. As shown in **Figure 3A**, eight genes, namely, BTK, IDO1, VAV1, TLR10, STAT4, SASH3, C3AR1, and RGS18, were considered the important nodes based on their log (FC) >1.5. In addition, GO biological process analysis and KEGG pathway analysis were performed based on these 55 HF-DEGs. The results showed that most of the biological processes were related to immunity, such as positive regulation of lymphocyte-mediated immunity (**Figures 3B, C** **)**, and the main KEEG pathway of these HF-DEGs was the NF-kappa B signaling pathway (**Figures 3D, E** **)**.

### The Correlation Between SASH3 Expression and Prognosis of Invasive Breast Cancer

To validate the results of the above bioinformatics analysis, we selected a gene named SASH3, one of the important nodes in the PPI network of HF-DEGs described above for further analysis. Consistent with our present data, SASH3 has been reported to be associated with prognosis of breast cancer by other bioinformatics analysis recently (9), but it has not been verified by further experiments. In this study, we first analyzed the correlation between SASH3 expression and prognosis of invasive breast cancer by using COX analysis. The clinical data of SASH3 were obtained from the TCGA database, and nine clinical pathological factors (ER status, PR status, HER2 status, AJCC stage, tumor size, number of lymph nodes, age (divided by 60), BRCA molecular subtypes, and SASH3 expression) were selected for COX analysis. Univariate analysis showed that three factors, including SASH3 expression, number of lymph nodes, and tumor size, were significantly associated with RFS in 467 invasive BRCA patients (*p* < 0.05, **Table 3**). Multivariate analysis including SASH3 expression, number of lymph nodes, tumor size, and AJCC stage (all *p* < 0.2) showed that SASH3 expression and number of lymph nodes were significantly associated with prognosis of invasive BRCA patients (*p* < 0.05, **Table 3**). Consistent with the RFS results obtained from the survival analysis of SASH3 expression described above (**Supplementary Table S3**), the results of both univariate analysis and multivariate analysis indicated that SASH3 expression was a favorable factor for prognosis of invasive BRCA (**Table 3**).

**Table 3:** The COX analysis of SASH3 in clinical data from the TCGA database.

Feature (categories)	RFS univariate analysis			RFS multivariate analysis		
	HR	95% CI	*p*-value	HR	95% CI	*p*-value
SASH3	0.44	0.203–0.952	0.032^*^	0.405	0.179–0.917	0.03^*^
Tumor size	1.39	1.001–1.941	0.049^*^	1.983	0.774–5.081	0.569
Lymph nodes	1.869	1.372–2.545	<0.001^***^	7.997	1.64–39.798	0.012^*^
AJCC stage	1.288	0.915–1.814	0.148	0.192	0.003–1.256	0.085
Age	1.114	0.599–2.071	0.732
ER	1.230	0.587–2.573	0.583
PR	0.803	0.433–1.488	0.486
HER2	0.553	0.171–1.790	0.316
Subtype	1.024	0.788–1.331	0.856

CI, confidence interval; HR, hazard ratio. ^*^p < 0.05, ^***^p < 0.001.

**Figure 4:**
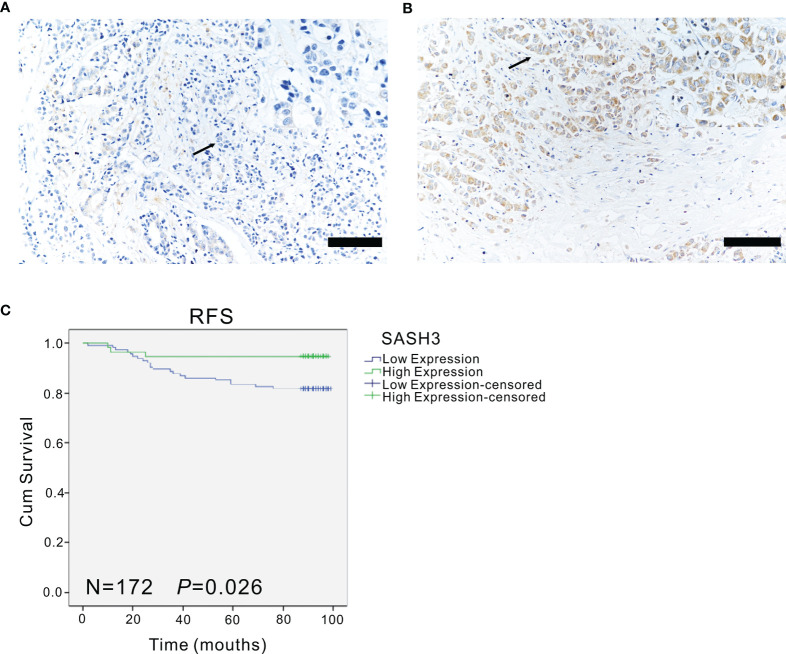
The expression of SASH3 in invasive breast cancer patients. **(A, B)** The representative immunohistochemical images for the low **(A)** and high **(B)** expression of SASH3 in human invasive breast cancer samples. Arrows indicated the magnified regions in the insert. Magnification: ×40. Scale Bars: 100 µm. **(C)** Survival curves showed the association of SASH3 (*n* = 172, *p* = 0.026) expression with the RFS in 172 human invasive breast cancer tissues.

Moreover, to verify the association of SASH3 protein expression with the prognosis of invasive BRCA, immunohistochemistry was performed. The results showed that high expression of SASH3 was observed in 57 (33.1%) patients, and low expression of SASH3 was observed in 115 (66.9%) patients. Survival analysis showed that high SASH3 expression was significantly associated with a longer RFS in invasive BRCA patients (*p* = 0.026, **Figure 4**). In addition, COX analysis of SASH3 based on the clinical information of these 172 invasive BRCA patients was performed. As shown in **Table 4**, univariate analysis showed that three factors, including SASH3 expression, number of lymph nodes, and AJCC stage, were significantly associated with RFS (*p* < 0.05, **Table 4**). Multivariate analysis showed that the number of lymph nodes was associated with poor RFS (*p* < 0.05, **Table 4**).

**Table 4:** The COX analysis of SASH3 in clinical data from the first affiliated hospital of china medical university.

Feature (categories)	RFS univariate analysis			RFS multivariate analysis		
	HR	95% CI	*p*-value	HR	95% CI	*p*-value
SASH3	0.276	0.082–0.926	0.037^*^	0.447	0.123–1.62	0.22
Lymph node	3.076	2.054–4.607	<0.001^***^	5.702	1.724–18.865	0.004^**^
AJCC stage	4.956	2.199–11.172	<0.001^***^	0.151	0.016–1.397	0.096
Age	0.977	0.439–2.175	0.955
ER	1.387	0.551–3.494	0.488
PR	0.949	0.421–2.136	0.899
HER2	0.553	0.171–1.790	0.316
Tumor size	1.349	0.402–4.524	0.627

CI, confidence interval; HR, hazard ratio. ^*^p < 0.05, ^**^p < 0.01, ^***^p < 0.001.

## Discussion

Tumor microenvironment contributes to the development, relapse, and therapy resistance of many cancers and is associated with prognosis of cancer patients (3). In the present study, we extracted a list of HF-DEGs related to the BRCA tumor microenvironment by functional enrichment analysis of the TCGA database and immune/stromal/ESTIMATE scores. The HF-DEGs identified here were significantly associated with the prognosis of invasive BRCA patients. Our findings suggest that the HF-DEGs may be potential key regulators for the tumor microenvironment of invasive BRCA and may represent novel biomarkers for the prognosis of invasive BRCA.

In this study, 55 tumor microenvironment-related HF-DEGs were identified in invasive BRCA. Of the 55 HF-DEGs, three genes, namely, DARC (10), CD40 (11), and DOK2 (12) have been previously reported to be associated with the better prognosis of BRCA patients when they were highly expressed. These reports further validated the accuracy of our approach. In addition, *in vitro* studies have shown that ten genes including CXCL10 (13), STAT4 (14), CD69 (15), C3 (16), ACSL5 (17), GZMH (18), TRIM22 (19), EMILIN2 (20), HTRA4 (21), and TSPAN2 (22) inhibited the proliferation, migration, and invasion or promoted apoptosis of BRCA cells, suggesting that the high expression of these genes may be correlated with a good prognosis, which were consistent with our present results. On the other hand, IL-1B, a HF-DEG identified in this study, has been previously identified as biomarker that could be used to predict which primary BRCA patients were likely to experience relapse in bone (23). It seems contrary to our results, probably due to the different types of BRCA, studied by other group and ours, respectively. Another eight genes including GBP1 (24), BTK (25), CXCL2 (26), IDO1 (27), FASLG (28), FIGF (29), PLAUR (30), and TNFRSF17 (31) were found to promote the migration, invasion, or proliferation of BRCA cells. Another two genes, namely, VAV1and RARRES1, were previously reported that they had pleiotropic results in BRCA. It was shown that knockdown of VAV1 inhibited the proliferation of the BRCA T47D cells (32), while overexpression of VAV1 inhibited the proliferation of the BRCA MDA-MB-231, MCF-7, and MDA-MB-453 cells (33). High RARRES1 expression was correlated with poor median survival of patients with inflammatory BRCA (34), while cell proliferation and tumor growth assays showed that RARRES1 was a tumor suppressor in triple-negative BRCA cell lines (35). These results seem contradictory, likely due to the use of different cell lines or different subtypes of BRCA. Moreover, bioinformatics analysis by other groups showed that six genes, including IRAK3, NCF4, HSD11B1, C3AR1, TLR10, and SASH3 were related to BRCA. Single nucleotide polymorphism (SNP) and gene-based analyses showed that the SNPs of IRAK3 (rs1732877) and NCF4 (rs1883113) may be associated with BRCA risk (36). Heather et al. discovered two SNPs in HSD11B1 (rs11807619, rs932335), which may increase risk for BRCA (37). Oncomine database analysis showed that C3AR1 was highly expressed in both primary and invasive ductal breast carcinoma (38). TCGA database analysis showed that TLR10 exhibited lower expression levels in advanced stages than that in earlier stages of BRCA (39). Whether these gene expressions are associated with prognosis of BRCA remains unknown. Further investigation is required to explore how these genes influence the development of BRCA and whether they can serve as biomarkers for prognostic prediction of BRCA. Strikingly, the remaining 25 genes, including ENPP2, HAVCR2, DPT, FAM30A, GIMAP8, GIMAP2, CXorf21, ATP8B4, MDFIC, GPR171, RUBCNL, WDFY4, SAMD3, FPRL2, RGS18, SUCNR1, FGL2, TMEM100, SLC7A7, IL18RAP, CST7, ADD3, RASGRP2, P2RY12, and DOCK2 have not been identified to be associated with the occurring, development, and prognosis of BRCA. Our findings suggest that they may be perceived as novel biomarkers for BRCA prognosis.

Recently, SASH3, one of the HF-DEGs identified here was also reported to be a tumor microenvironment-related gene with prognostic value in BRCA by other bioinformatics analysis (9). To further validate our results of HF-DEGs with prognostic value in invasive BRCA, we determined the correlation of SASH3 with the prognosis in invasive BRCA patients by using COX analysis and immunohistochemical analysis. The results showed that high SASH3 expression was positively correlated with longer RFS in invasive BRCA patients, suggesting that SASH3 expression may be used as an independent prognostic indicator for invasive BRCA patients. SASH3 contains Src homology 3 (SH3) and sterile alpha motif (SAM) domains, which are involved in many cell signaling transduction pathways. It has been reported that SASH3 functions as an adaptor protein in lymphocytes (40–42). Recent study showed that SASH3 is important for T-cell proliferation, activation and cell survival, and lack or mutation of SASH3 could lead to a new type disease of human X-linked combined immunodeficiency, which was manifested as CD4^+^ T-cell lymphopenia, decreased T-cell proliferation, cell cycle progression, and increased T-cell apoptosis in response to mitogens (43). However, the precise function of SASH3 remains unknown. The role of SASH3 in tumorigenesis and development has not been reported yet, which warrants future investigation. SASH1, containing highly similar protein structure with SASH3, functions as a tumor suppressor to inhibit the development of many cancers, such as colon cancer, gastric cancer, BRCA, and cervical cancer (44–47). Therefore, we speculate that SASH3 may serve as a tumor suppressor to inhibit the occurrence and development of invasive BRCA. Further exploration of the function of the HF-DEGs identified in this study may provide new strategies for diagnosis, treatment, and prognosis of invasive BRCA.

In recent years, great progress has been made in predicting the correlation between invasive BRCA prognosis and gene expression. Many of these studies were conducted by the construction of animal models, cell experiments *in vitro*, and small-scale studies of clinical tumor samples. However, a large-scale comprehensive analysis is still required for exploring the complex interactions between invasive BRCA and its tumor microenvironment. In this study, by deeply mining TCGA database, we provided a comprehensive analysis of the prognostic impact of the tumor microenvironment-related genes in invasive BRCA. Moreover, functional enrichment analysis suggested that the tumor microenvironment-related HF-DEGs identified here were mainly involved in immune responses, which provided novel insights into understanding the underlying mechanisms of the HF-DEGs in invasive BRCA.

In conclusion, we identified a list of HF-DEGs which were positively correlated with good prognosis in invasive BRCA patients. In-depth study of these HF-DEGs may lead to a deeper understanding of the tumor microenvironment of invasive BRCA and provide more guidance for the clinical diagnosis, treatment, and prognosis of invasive BRCA. Moreover, our gene mining strategy related to the tumor microenvironment can be widely applied in big data analysis and to find more accurate and reliable biomarkers with prognostic values for other malignant tumors.

## Data Availability Statement

The original gene expression data (AgilentG4502A expression data) and relevant clinical information that support the finding of this study were obtained from the TCGA database (https://tcga-data.nci.nih.gov/tcga/). The immune/stromal/ESTIMATE scores (The scores were calculated based on AgilentG4502A expression data) of invasive BRCA were downloaded from https://bioinformatics.mdanderson.org.

## Ethics Statement

The studies involving human participants were reviewed and approved by the Scientific Ethical Committee of China Medical University (No. 2017066). Written informed consent for participation was not required for this study in accordance with the national legislation and the institutional requirements.

## Author Contributions

YF and FY: conceptualization. YS, SC, HX, FJ, and QX: methodology. YS, HX, and NW: software. YS, SC, GJ, and QL: validation. YS, SC, HX, and NW: formal analysis. YS and SC: investigation. NS, TK, RS, FJ, and FY: resources. YS and SC: date curation. YS, YF, and FY: writing—original draft. YS, SC, GJ, and YF: writing—review and editing. YS and SC: visualization. YF and FY: supervision. YF and FY: project administration. YF, SC, and FY: funding acquisition. All authors have read and agreed to the published version of the manuscript.

## Funding

This work was supported by grants from the National Natural Science Foundation of China, China (No. 81974418) and Basic Research Project of Liaoning Province, China (No. JC2019002) to FY. This work was also supported by research fund from Doctoral Start up Foundation of Liaoning Province, China (No. 2019-BS-284) and Youth backbone Support Program of China Medical University, China (No. QGZ2018081) to SC. The funders had no role in study design, data collection and interpretation, or the decision to submit the work for publication.

## Conflict of Interest

The authors declare that the research was conducted in the absence of any commercial or financial relationships that could be construed as a potential conflict of interest.

## Publisher’s Note

All claims expressed in this article are solely those of the authors and do not necessarily represent those of their affiliated organizations, or those of the publisher, the editors and the reviewers. Any product that may be evaluated in this article, or claim that may be made by its manufacturer, is not guaranteed or endorsed by the publisher.

## Supplementary Material

Supplementary Table 1 The DEGs of the four important modules.

Supplementary Table 2 The P value of the 392 DEGs calculated by Kaplan-Meier Plotter kmplot.com.

Supplementary Table 3 The P value of the 55 HF-DEGs calculated by R and Kaplan-Meier Plotter kmplot.com.

Supplementary Figure 1 The main biological processes in which the DEGs of each module were involved.

Supplementary Figure 2 The main KEGG pathways in which the DEGs of each module were involved.

Supplementary Figure 3 The RFS curves of the 61 DEGs in TCGA database calculated by R.
