# Supplementary material for: Comprehensive Analysis of Prognostic Microenvironment-Related Genes in Invasive Breast Cancer
Source: Front Oncol. 2022 Jan 3;11:576911. doi: 10.3389/fonc.2021.576911 (PMC8761742; doi:10.3389/fonc.2021.576911)
Supplement: Supplementary Table 1 — The DEGs of the four important modules. [file Table_1.docx]

**Table S1. The DEGs of the four important modules.**

| Module | Nodes | Edges | DEG IDs |
| --- | --- | --- | --- |
| 1 | 34 | 378 | ITGA4, FCGR2B, KIT, IL6, IL1B, CD69, CCL2, IDO1, SLAMF1, CCL11, TBX21, CD33, FAS, FASLG, KLRB1, IL7, IL7R, VCAM1, HAVCR2, CD28, TNFSF13B, TNFSF11, IL33, SELP, TLR8, CD19, CD38, SELE, TLR4, CD40, **PTPRC,** GPR29, PDCD1LG2, CD1C |
| 2 | 24 | 261 | GPR18, S1PR1, P2RY12, P2RY13, CCL19, SUCNR1, P2RY14, CCR2, GNG2, **CXCL2,** TLR7, C3, PNOC, SAA1, FPR3, GPR183, ANXA1, CCL13, CXCR6, CXCL12, S1PR4, CXCL11, CXCR3, C3AR1 |
| 3 | 48 | 285 | CD247, IL16, APBB1IP, ITK, CCR7, NCKAP1L, IKZF1, CD52, OLR1, HVCN1, CD27, STAT4, CD74, FYN, EOMES, CD36, FERMT3, KLRD1, NLRP3, TLR10, CASP1, LAIR1, CD53, HCLS1, BST1**, CD48,** CYBB, PARVG, CD5, TNFRSF1B, CD2, ICOS, PLAUR, PLAU, EVI2B, MME, SELPLG, CD1E, ATP8B4, PRDM1, PLEK, LCK, IL21R, SASH3, DOCK2, TLR3, GMFG, CD3D |
| 4 | 27 | 133 | XCL2, **ITGB2,** NCF4, WDFY4, CYTIP, FYB, ALOX5AP, SH2D1A, BTLA, CORO1A, LCP2, VAV1, RGS18, CD3G, SLA, TRAT1, RASAL3, DOCK8, AIF1, LAPTM5, CD79B, LY86, TAGAP, MNDA, RNASE6, CLEC10A, ARHGAP30 |

* Genes in bold are the remarkable genes for each module. ****P* < 0.0001.
