# Supplementary figures and images for: Comprehensive Analysis of Prognostic Microenvironment-Related Genes in Invasive Breast Cancer
Source: Front Oncol. 2022 Jan 3;11:576911. doi: 10.3389/fonc.2021.576911 (PMC8761742; doi:10.3389/fonc.2021.576911)

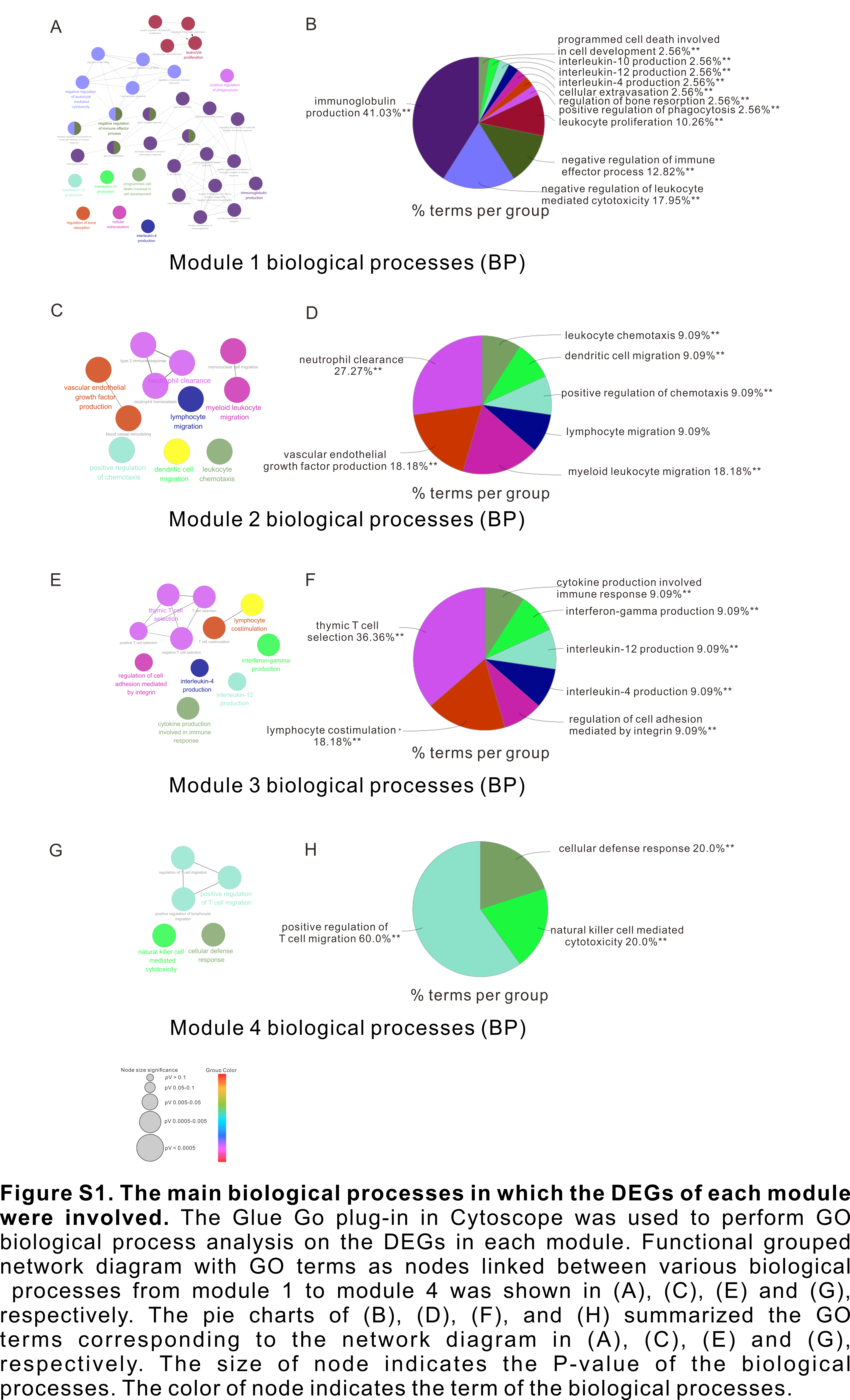

Supplement: Supplementary Figure 1 — The main biological processes in which the DEGs of each module were involved. [file Image_1.tif]

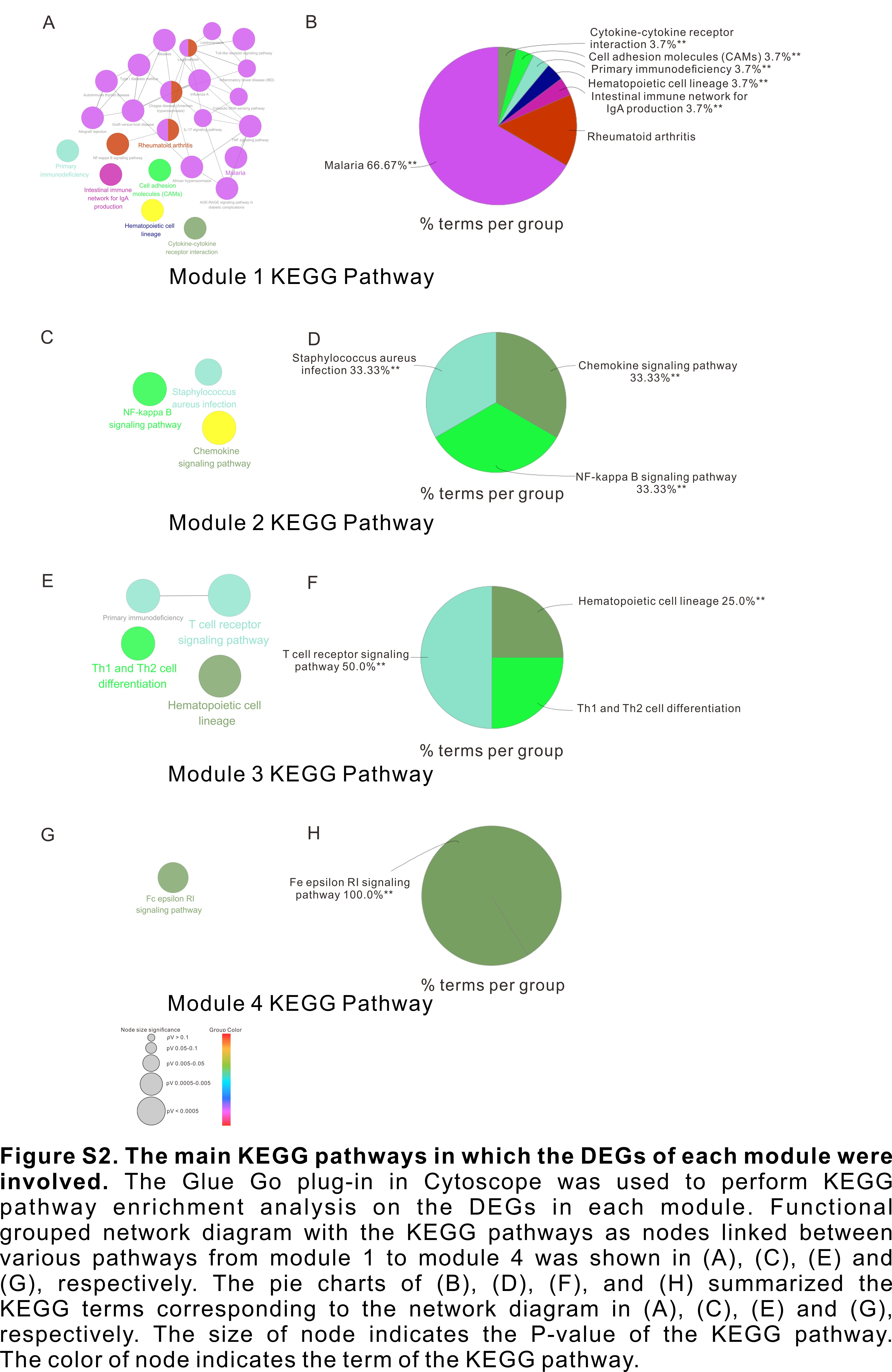

Supplement: Supplementary Figure 2 — The main KEGG pathways in which the DEGs of each module were involved. [file Image_2.tif]

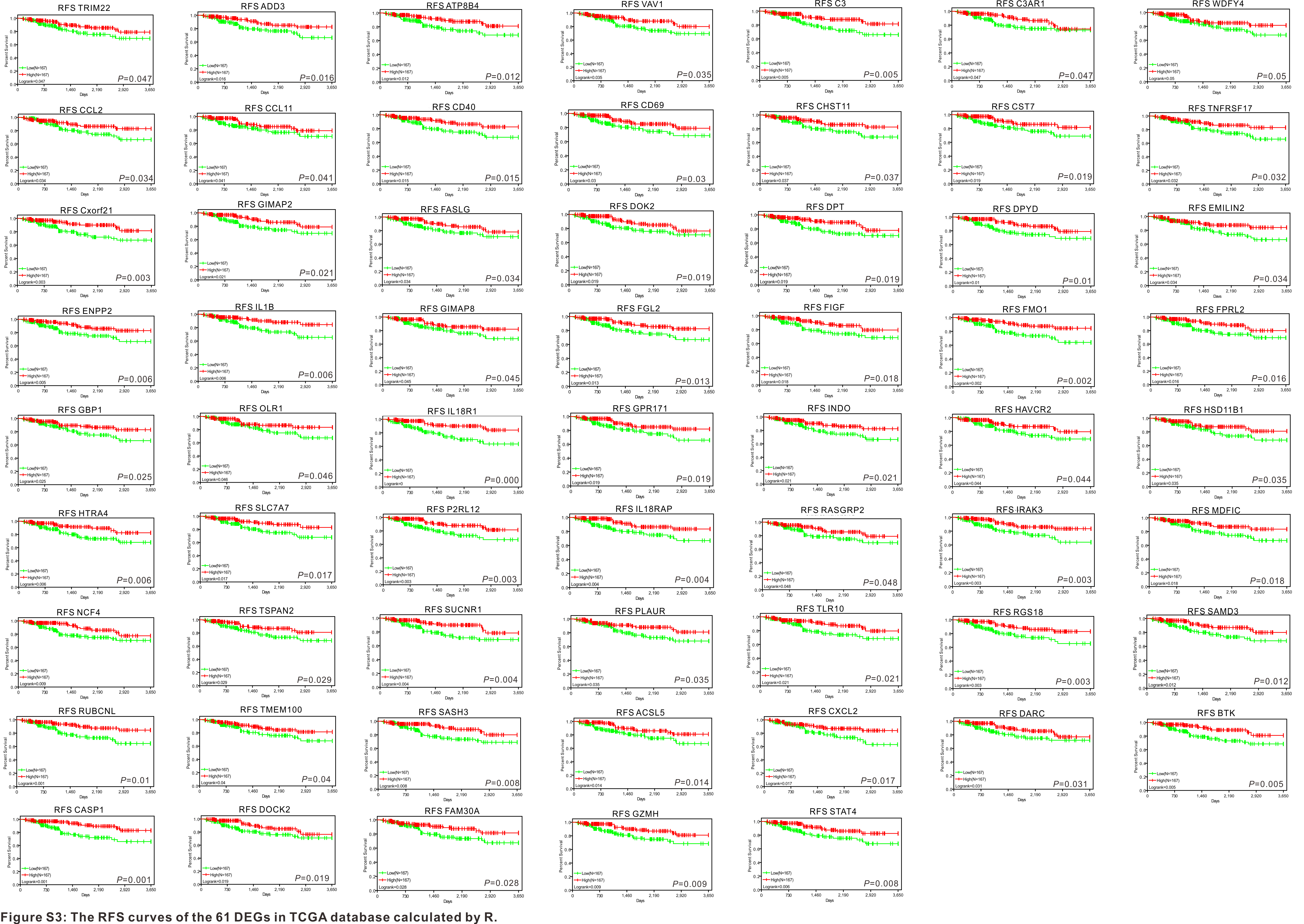

Supplement: Supplementary Figure 3 — The RFS curves of the 61 DEGs in TCGA database calculated by R. [file Image_3.tif]
